# Measurement of Two-Photon Absorption Cross Section of Metal Ions by a Mass Sedimentation Approach

**DOI:** 10.1038/srep17712

**Published:** 2015-12-11

**Authors:** Zhuo-Chen Ma, Qi-Dai Chen, Bing Han, Xue-Qing Liu, Jun-Feng Song, Hong-Bo Sun

**Affiliations:** 1 State Key Laboratory on Integrated Optoelectronics, College of Electronic Science and Engineering, Jilin University, 2699 Qianjin Street, Changchun, 130012, People’s Republic of China; 2 College of Physics, Jilin University, 119 Jiefang Road, Changchun, 130023, People’s Republic of China

## Abstract

The photo-reduction of metal ions in solution induced by femtosecond laser is an important and novel method for fabricating three-dimensional metal microstructures. However, the nonlinear absorption cross section of metal ions remains unknown because its measurement is difficult. In the present study, a method based on Two-Photon Excited Sedimentation (TPES) is proposed to measure the two-photon absorption cross section (TPACS) of metal ions in solution. The power-squared dependence of the amount of sediment on the excitation intensity was confirmed, revealing that 800 nm femtosecond laser induced reduction of metal ions was a two photon absorption process. We believe that the proposed method may be applied to measure the TPACS of several metal ions, thereby opening a new avenue towards future analysis of two-photon absorption materials.

Since the phenomenon of two-photon absorption (TPA) was first experimentally observed in 1961^1^, it has attracted much research interest because of its potential application in three-dimensional optical data storage^2^,^3^,^4^,^5^, two-photon fluorescence imaging^6^,^7^,^8^, and microfabrication^9^,^10^,^11^,^12^,^13^. The femtosecond laser two-photon fabrication technique, which utilizes TPA, has become widely used in various fields^10^,^12^,^14^,^15^, among which the TPA induced reduction of metal ions^15^,^16^,^17^,^18^ is of great significance due to their unique properties of metal nanostructures in various applications such as surface enhanced Raman scattering^19^, catalytic reactions^20^, optical sensing^21^, and metal nanowiring for electronic interconnections^22^. Up to now, however, the two-photon absorption cross section (TPACS) of metal ions, which characterizes the capability of TPA, still remains unknown because of the difficulty to measure it, since the existing methods for studying TPA are not applicable to metal ions. Thus far, several techniques have been adopted to determine the TPACS of various materials, among which two methods are the most commonly used: Two Photon Excited Fluorescence (TPEF)^23^,^24^,^25^ and Z-scan^26^,^27^,^28^,^29^, neither of which is applicable to metal ions. The absence of fluorescence emission in most metal ions precludes the use of TPEF method for measuring the TPACS of metal ions. Z-scan measurement^30^,^31^ is widely used to measure non-linear refractive index and non-linear absorption coefficient via the “closed” and “open” methods respectively. In this measurement, the sample is typically placed at the focus of a lens, and then moved along the z axis. But Z-scan technique cannot be applied to measure the TPACS of metal ions because of the following reasons: (1) Metal ions in solution would be photo-reduced to neutral metal atoms upon laser irradiation and then aggregate into metal particles, which lead to severe scattering. (2) The TPACS of metal ions is much smaller than the detection limit of the Z-scan technique. Therefore, there exists an urgent need for a new approach to the measurement of TPACS of metal ions.

In the present study, we propose a new method based on mass sedimentation for measuring the TPACS of metal ions at the excitation wavelength of 800 nm pulsed laser. When irradiated by excitation laser, metal ions are reduced to neutral atoms, thus precipitating from solution. By measuring the variation in metal ion concentration after irradiation using an 800 nm femtosecond laser (TPA) and a 405 nm continuous wave (CW) laser (single-photon absorption; SPA), we can compare the amounts of sediment under these two conditions. Further, the absorbed photon numbers of 800 nm laser could be derived through comparison with single-photon excitation (405 nm CW laser), assuming that single-photon and two-photon excitation have the same sedimentation efficiency. In this manner, the TPACS of metal ions at 800 nm excitation can be calculated. We also tested the power squared dependence of two-photon excited sedimentation on the laser intensity for metal ions, demonstrating that the reduction of metal ions induced by 800 nm femtosecond laser was a TPA process.

## Results and Discussion

**Figure 1:**
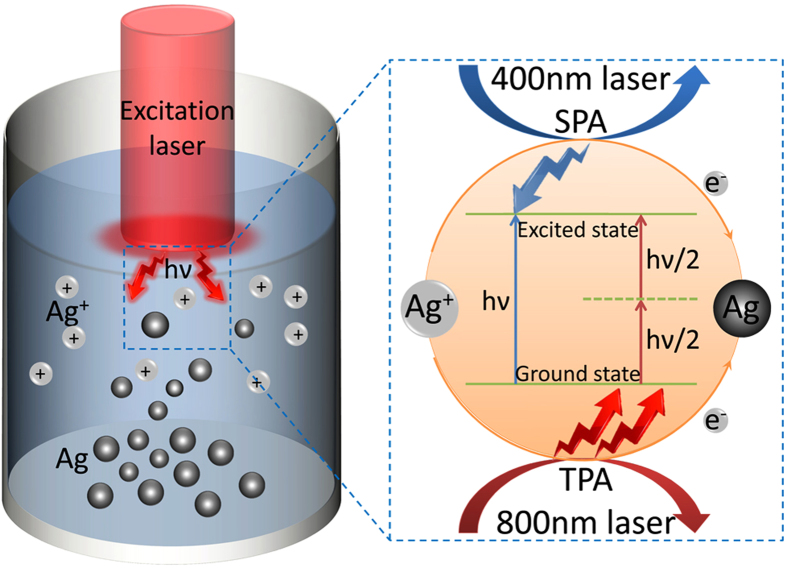
Schematic for measuring two-photon absorption cross section of metal ions. Here 400 nm CW laser and 800 nm pulsed femtosecond laser are adopted for single-photon absorption (SPA) and two-photon absorption (TPA) respectively, resulting in mass sedimentation of neutral silver atoms.

Metal ions can be reduced to neutral atoms when excited by both SPA and TPA, leading to the formation of metal sediment (Fig. 1). By taking the silver ion as an example, Ag^+^ could be excited by both 405 nm CW laser and 800 nm pulsed laser, followed by electron capture from the surrounding medium, resulting in the formation of a silver atom Ag^0^. The subsequent agglomeration process of Ag^0^ precipitates silver nanoparticles from solution containing silver ions, namely mass sedimentation. As is well known, from the aspect of TPA definition, it is equivalent for a molecule to be excited from ground state to a higher energy state by simultaneous absorption of two photons with low energy and by absorption of one photon with high energy. Therefore, in our experiment, if the concentration of silver ions in two identical solutions changes by the same amount under conditions of SPA and TPA, the number of photons absorbed in TPA (N_TPA_) will be twice that in SPA (N_SPA_). That is, N_TPA_ should be proportional to N_SPA_, which can be expressed as follows:


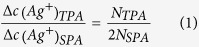


where Δc(Ag^+^)_TPA_ and Δc(Ag^+^)_SPA_ represent the variation in silver ion concentration under TPA and SPA respectively. Therefore, through a comparison between TPA and SPA, the absorbed photon numbers could be derived, enabling the calculation of the TPACS of silver ion. In this process, we make the assumption that single-photon excitation and two-photon excitation have the same sedimentation efficiency^23^.

**Figure 2:**
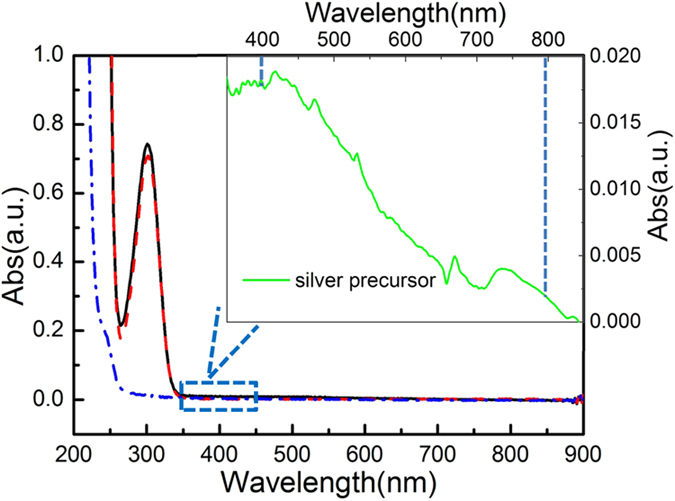
Optical absorption spectrum of 0.1 M silver ion precursor solution and its constituents. The solid curve represents 0.1 M silver precursor solution, the dash curve represents 0.1 M silver nitrate solution, and the dash dot curve represents sodium citrate aqueous solution. Inset shows the locally magnified absorption spectrum around 400 nm and 800 nm.

In our work, two systems were adopted to investigate the SPA and TPA of silver ion in the precursor solution. The excitation source for TPA was a Spectra-Physics Ti: sapphire amplifier laser system. The laser output had a central wavelength of 800 nm and pulse duration of 100 fs at a repetition rate of 1 kHz. The light source for SPA was a semiconductor laser with an output wavelength of 405 nm. Our sample solution for photo-reduction of silver was composed of silver ammonia complexation ion and sodium citrate^22^. The silver ion precursor solution was prepared by dropping a suitable amount of aqueous ammonia into a mixture of silver nitrate (0.1 M) and trisodium citrate (0.1 M) aqueous solution under stirring until a clear solution was formed. Figure 2 shows the optical absorption spectra of a pure silver nitrate aqueous solution, a pure sodium citrate solution, and the mixture of silver ammonia complexation ion and sodium citrate solution (sample solution) used for femtosecond laser induced reduction. An absorption band with a peak at approximately 302 nm is observed in the spectra for both the pure silver nitrate solution and the mixture solution, but it is absent in the sodium citrate solution, indicating that this absorption band originates from the silver ion. In the entire spectra, no obvious absorption can be observed at the excitation wavelength of 800 nm (OD ≤ 10^−3^), while the inset shown in Fig. 2 demonstrates weak absorption at 405 nm (OD = 0.017), indicating that the reduction of silver ions induced by 800 nm femtosecond laser should be a TPA process. By utilizing Ti: Sapphire femtosecond laser oscillator for direct writing, we could produce arbitrary silver patterns on substrates as a consequence of the TPA induced reduction of silver ions, as shown in Fig. S1 (pigeon pair and plane). Furthermore, no metal precipitate could be observed when mode locking of the 800 nm Ti: Sapphire laser was turned off, proving that the photo-reduction of silver ion was a TPA process. Actually, in our experiment, which employs both 405 nm CW laser and 800 nm pulsed laser, silver precipitates could be simultaneously formed, while using an 800 nm CW laser did not produce any silver sediment. All these phenomena further corroborated our belief that the photo-reduction of silver ion with an 800 nm pulsed laser was a TPA process^15^.

**Figure 3:**
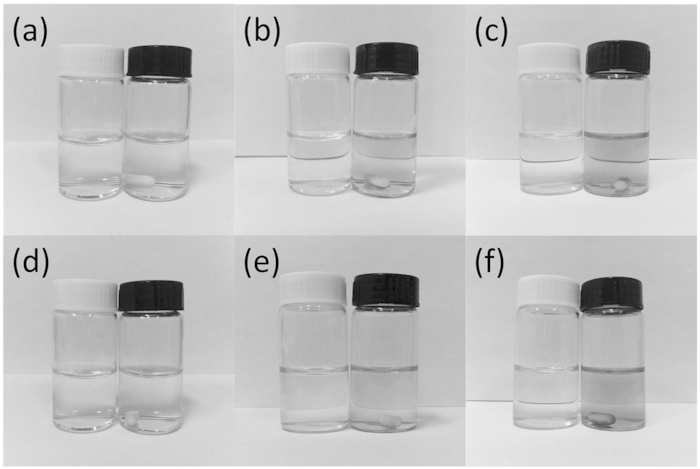
Evolution of the solution color during the formation of silver nanoparticles when irradiated by 405 nm CW laser (a–c) and 800 nm femtosecond laser (d–f). (**a,d**) Irradiation time 1 h; (**b,e**) Irradiation time 2 h; (**c,f**) Irradiation time 3 h. In each photograph, the left and right vials correspond to the non-irradiated and irradiated solution respectively.

**Figure 4:**
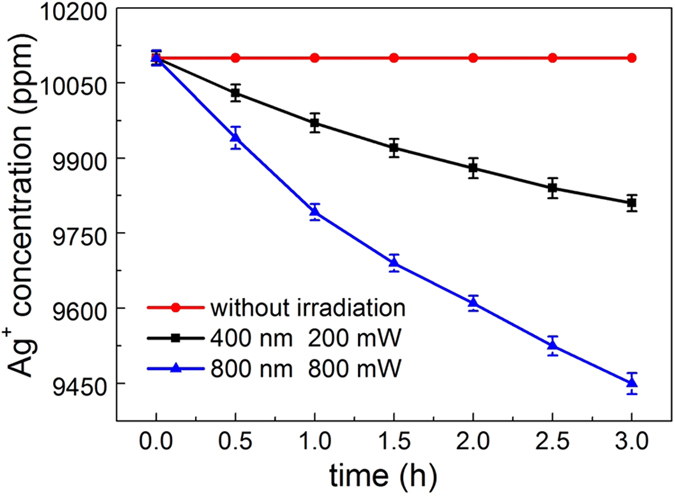
Variation of the concentration of silver ion with proceeding irradiation time. The circle point curve represents the sample without laser irradiation; the square point curve represents the sample under 405 nm laser irradiation with the power of 200 mW; the triangular point curve represents the sample under 800 nm laser irradiation with the power of 800 mW.

The 405 nm and 800 nm excitation lasers were introduced into our sample solution through a reflection mirror vertically from top to bottom, while simultaneously, a small stirrer was placed in the sample solution for adequately stirring under the control of a magnetic stirring apparatus. In this way, the formation of a silver mirror on the surface of the sample solution could be avoided, ensuring that excitation laser can transmit through the solution from top to bottom. Both of the laser spot diameters were adjusted to be 5 mm by using an aperture. In our experiment, the power of the 405 nm laser was set to be 200 mW, which was measured after the aperture, while that of 800 nm laser was varied by use of a ND filter. After having been irradiated by excitation laser, the original colorless aqueous solution started to deepen in color with increasing irradiation time, revealing the formation of silver nanoparticles (Fig. 3). Figure 3a–c demonstrate that the originally clear silver ion precursor solution became increasingly turbid because of the 405 nm laser irradiation. Figure 3d–f show this gradual change under irradiation of 800 nm laser. These photos were recorded from irradiation time of 1 hour to 3 hours at intervals of 1 hour. In the photographs, the sample on the right is the one irradiated by either 405 nm laser (Fig. 3a–c) or 800 nm laser (Fig. 3d–f), while that on the left is the non-irradiated sample kept in the dark, serving as a contrast. Apparently, no color change was observed for the non-irradiated solution. After the sample solution with a large number of suspended silver nanoparticles was centrifuged, silver nanoparticles precipitated into the bottom of sample cell, leaving upper solution clear. By taking out of the upper clear solution, we measured its silver concentration through atomic absorption spectrometry (PinAAcle 900T, PerkinElmer, USA). Figure 4 shows the gradual change in the concentration of silver ion under 405 nm and 800 nm laser irradiation. Note that the concentration of silver ion remains unchanged throughout the process if the solution was kept in the dark without irradiation. The initial silver ion concentration of 10100 ppm, decreased with proceeding irradiation time under both 405 nm and 800 nm laser irradiation. We can also separate the silver nanoparticle precipitates prepared by laser irradiation and then characterize them using SEM and X-ray diffraction (XRD) which are shown in Figs S2 and S3.

It is well known that the two photon absorption coefficient is defined by


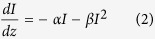


where I is the laser intensity, z is the direction of light propagation, α is the linear absorption coefficient, and β is the TPA coefficient. From this equation, we can conclude that when TPA appears, α = 0, and


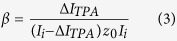


where ΔI_TPA_ is the absorption laser intensity of the sample solution on occasion of TPA, I_i_ is the input laser intensity, and z_0_ is sample length. With a fixed input laser intensity I_i_, in order to calculate the TPA coefficient β, we only need to determine ΔI_TPA_, which can be obtained using Eq. (1). If we make a comparison of Δc (Ag^+^) between SPA and TPA, e.g., for the group of data with both SPA and TPA at an irradiation time of 1 h, Δc (Ag^+^)_TPA_ = 2.37 Δc (Ag^+^)_SPA_, considering that the 800 nm femtosecond laser for TPA is pulsed with a repetition frequency of 1 kHz and a duration of 100 fs, therefore ΔI_TPA_ = 2.37 × 10^10^ ΔI_SPA_. According to the Beer’s law for linear absorption,


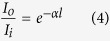


where I_o_ is the output light intensity, I_i_ is the input light intensity, α is the SPA coefficient, and l is the sample length. Therefore, ΔI_SPA_ = I_i_ − I_o_ = I_i_ (1–e^−αl^), here α can be calculated from the OD value of the sample solution at the wavelength of 405 nm, which is 0.017 cm^−1^, l is the sample length, which is 2 cm. Then, we can obtain ΔI_SPA_ = 346.53 W/m^2^, and ΔI_TPA_ = 8.21 × 10^12^ W/m^2^. In Eq. (3), the sample length z_0_ = 2 cm, and I_i_ which is the input intensity of 800 nm laser can be calculated as follows: as the average power of pulsed femtosecond laser was 800 mW, the peak intensity was thus actually 800 × 10^10^ mW, considering that the femtosecond laser was pulsed with a repetition rate of 1 kHz and a pulse width of 100 fs, and the laser spot was adjusted to be 5 mm in diameter, resulting in a laser spot area of π* (2.5 mm)^2^, so the laser peak intensity was 800 × 10^10^ mW/ [π* (2.5 mm)^2^], which was equal to 4 × 10^14^ W/m^2^. Thus, we can calculate that β is approximately 2.6 × 10^−4^ cm/GW. The TPACS of silver ion δ can be obtained from the following equation:


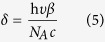


where N_A_ is the Avogadro constant and c is the silver ion concentration. After calculation, δ is about (9.97 ± 0.79) × 10^−2^ GM by averaging the values of several times of measurements.

**Figure 5:**
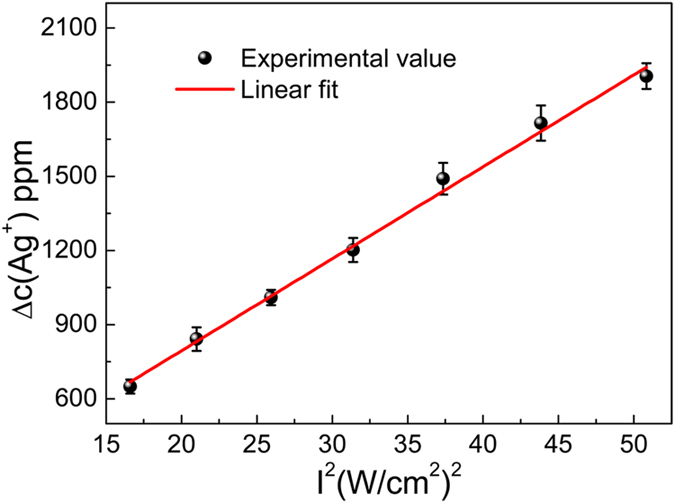
Dependence of two-photon induced variation of silver ion concentration on excitation intensity. The straight line is the linear fit.

Based on the above results, we tested the power-squared dependence of TPA induced mass sedimentation of silver ion. The variation in silver ion concentration Δc (Ag^+^) under different laser peak intensities is shown in Fig. 5. Excitation powers ranging from 0.8 W to 1.4 W with intervals of 0.1 W were tested. In this test, somehow, small deviations from the square-law dependence of TPA were observed, which might be due to the instability of laser output power and the instrumental error of atomic absorption spectroscopy. Provided that this small departure is acceptable, we could observe a nearly perfect power-squared dependence of TPA induced mass sedimentation of silver ion. The linear relationship showed that the femtosecond laser induced silver ion reduction was indeed caused by TPA.

In order to explore the underlying mechanism of TPA induced mass sedimentation of silver ion, we consider that, in former work, the irradiation of aqueous AgClO_4_ solution with UV light induced photo-oxidation of H_2_O by excited Ag^+^ , resulting in the formation of Ag^0^, H^+^, and OH radicals^32^,^33^,^34^,^35^. The subsequent agglomeration process of Ag^0^ produced colloidal silver, (Ag^0^) _n_, and bulk silver, Ag^M^ . This procedure is illustrated as follows:


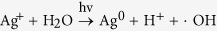



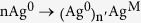


Therefore, in our work, we propose that silver ions could also be excited by TPA at the excitation wavelength of 800 nm, leading to excited oxidative Ag^+*^ which could further capture electrons from surrounding environment such as the reductant (trisodium citrate here). Consequently, the whole reactions included in this TPA induced reduction of silver ions are summarized as follows:


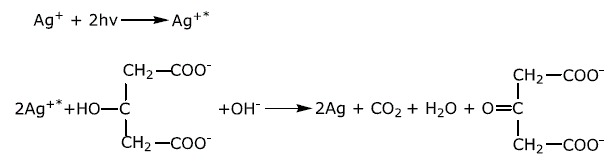


Compared with the previously established TPACS measurement method, our new TPES method has unique advantages, including: (a) Light scattering caused by the production of metal particles under excitation laser irradiation has little effect on metal sedimentation. This new TPES method only needs information on the amount of metal sediment. In contrast, for Z-scan technique, scattering has a serious impact on the transmitted light signal. (b) This new method is especially suitable for samples possessing small TPACS because of the “signal integration” effect. In the case of Z-scan technique, due to the small absorption of metal ions, variation in the transmitted light can hardly be observed, leading to a low signal-to-noise ratio or almost no useful signal. Yet, in the TPES method, the amount of metal sediment is an integration result of accumulated absorption which seems like an accumulation of weak transient absorption. Therefore, this method is expected to become a powerful tool for measurement of samples with small TPACS.

## Conclusion

We have put forward a new method based on mass sedimentation by comparing the SPA and TPA processes for measuring the TPACS of metal ions. The TPACS of silver ion at the excitation wavelength of 800 nm was measured to be approximately (9.97 ± 0.79) × 10^−2^ GM. The amount of sediment was proven to be proportional to the square of laser intensity, confirming that the photo-reduction of silver ion induced by 800 nm femtosecond laser was a TPA process. We believe that this newly developed method TPES may be applied to other metal ions as well, therefore opening a new avenue towards future analysis of TPA materials.

## Methods

Preparation of metal ion precursor solution: The silver ion precursor solution was prepared by dropping a suitable amount of aqueous ammonia into a mixture of silver nitrate (0.1 M) and trisodium citrate aqueous solution under stirring until a clear solution was formed. The optical absorption spectra were measured using a UV-VIS spectrophotometer (UV-2550, SHIMADZU).

Two-photon laser direct writing of silver pattern: A Ti: Sapphire laser system (Spectra-Physics Tsunami) with an operating wavelength of 800 nm, a pulse width of 100 fs, and a repetition frequency of 80 MHz was used as a light source. The laser beam was introduced to an inverted microscope and tightly focused at the interface between the silver ion solution and a cover slip by using an oil-immersion objective lens (100×, NA = 1.42). After the direct writing of silver pattern, the substrate was rinsed with deionized water and then dried under ambient environment. The surface morphologies of the samples were measured using a JEOL JSM-6700F field emission scanning electron microscope operating at 5.0 kV.

Irradiation of metal ion precursor solution: Excitation lasers of 400 nm and 800 nm were vertically introduced into our sample solution through a reflection mirror from top to bottom, while at the same time a small stirrer was placed in the sample solution for adequately stirring under the control of a magnetic stirring apparatus. In every experiment, a 5 ml sample solution was placed in a vial. Both of the laser spot diameters were adjusted to be 5 mm by an aperture. After irradiation by the excitation laser, the sample solution containing large numbers of suspended silver nanoparticles was centrifuged, consequently silver nanoparticles precipitated into the bottom of the sample cell, leaving a clear upper solution. After taking out of the clear upper solution, we measured its silver concentration using atomic absorption spectrometry (PinAAcle 900 T, PerkinElmer, USA). For XRD test, we separated the precipitates of silver nanoparticles produced by irradiation. XRD data was recorded on a RigakuD/Max-2550 diffractometer with Cu Kα radiation (λ = 0.15418 nm).

## Additional Information

**How to cite this article**: Ma, Z.-C. *et al.* Measurement of Two-Photon Absorption Cross Section of Metal Ions by a Mass Sedimentation Approach. *Sci. Rep.* **5**, 17712; doi: 10.1038/srep17712 (2015).

## Supplementary Material

Supplementary Information
