# Supplementary material for: Measurement of Two-Photon Absorption Cross Section of Metal Ions by a Mass Sedimentation Approach
Source: Sci Rep. 2015 Dec 11;5:17712. doi: 10.1038/srep17712 (PMC4675988; doi:10.1038/srep17712)
Supplement: Supplementary Information [file srep17712-s1.doc]

Measurement of Two-Photon Absorption Cross Section of Metal Ions by a Mass Sedimentation Approach

Zhuo-Chen Ma^1^, Qi-Dai Chen^1^, Bing Han^1^, Xue-Qing Liu^1^, Jun-Feng Song^1^, and Hong-Bo Sun*^1, 2^

^1^ State Key Laboratory on Integrated Optoelectronics, College of Electronic Science and Engineering, Jilin University, 2699 Qianjin Street, Changchun, 130012, People’s Republic of China.
^2^ College of Physics, Jilin University, 119 Jiefang Road, Changchun, 130023, People’s Republic of China.

E-mail: [hbsun@jlu.edu.cn](mailto:hbsun@jlu.edu.cn)

**Supplementary Information**

**Silver patterning by TPA induced reduction**


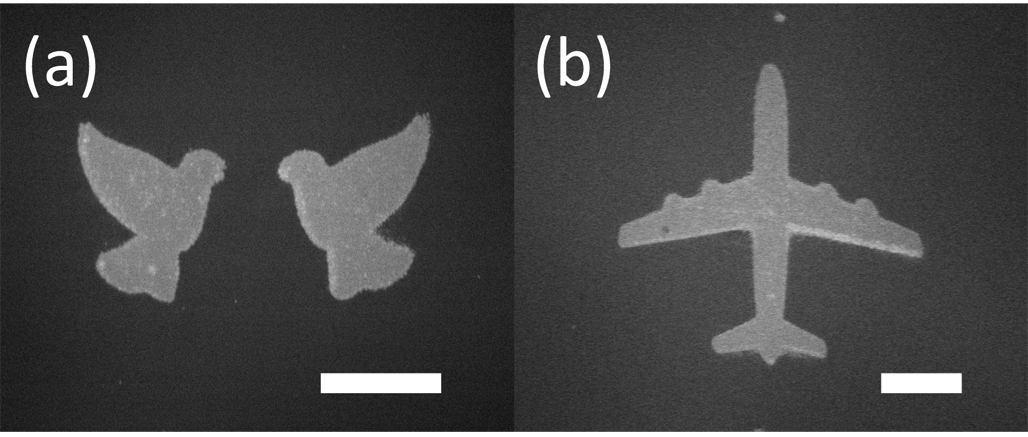


**Figure S1.** SEM images of silver patterns fabricated by two-photon induced reduction, micro-pigeon and micro-plane respectively. Scale bar is 10 μm.

Fig. S1 shows the femtosecond laser patterning of silver caused by TPA induced reduction of silver ions. Almost arbitrary patterns could be produced by this TPA process.

**Silver nanoparticles produced by laser irradiation**


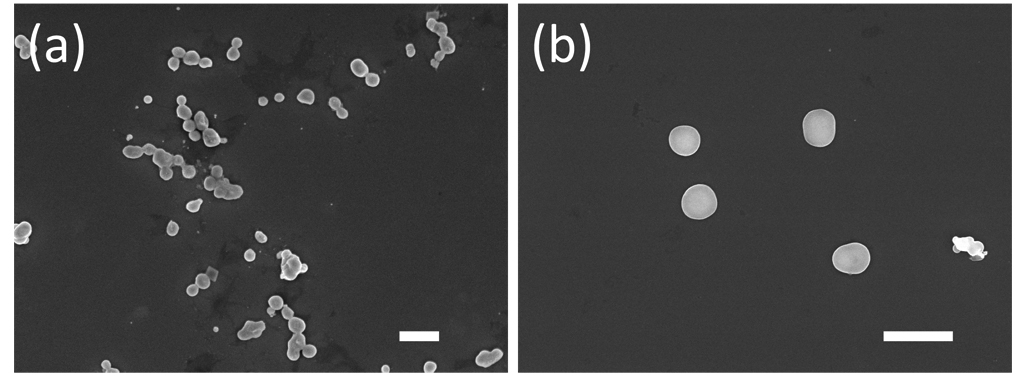


**Figure S2.** (a) SEM images of silver precipitates, scale bar: 1μm. (b) Locally magnified image of (a), scale bar: 1 μm.

Fig. S2 shows that silver nanoparticles could be produced upon laser irradiation. These precipitates were separated from the irradiated precursor solution after centrifuging and then dropped onto a silicon wafer for SEM characterization. The diameters of the as-precipitated silver nanoparticles were approximately 400-500 nanometers.

**XRD spectrum of silver precipitates**


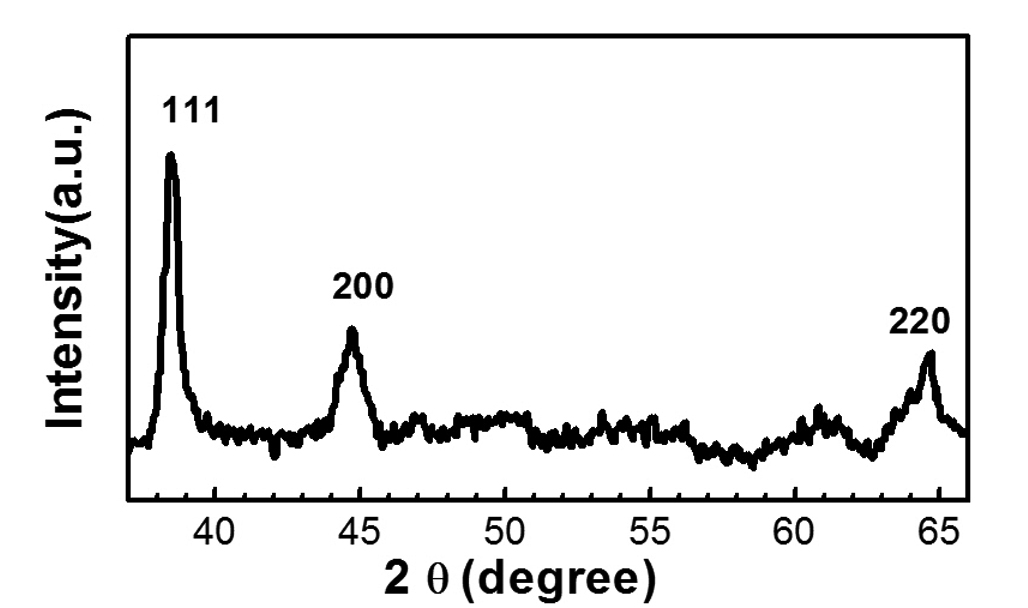


**Figure S3.** XRD spectrum of silver nanoparticles obtained from the precipitates after being irradiated by 800nm femtosecond laser.

Fig. S3 shows the XRD spectrum of the silver precipitates which indicates three distinct silver diffraction peaks related to (111), (200), and (220) planes.
